# Supplementary material for: Mechanisms governing the pioneering and redistribution capabilities of the non-classical pioneer PU.1
Source: Nat Commun. 2020 Jan 21;11:402. doi: 10.1038/s41467-019-13960-2 (PMC6972792; doi:10.1038/s41467-019-13960-2)
Supplement: Supplementary file 7 — Source data [file 41467_2019_13960_MOESM7_ESM.zip › Source_Data/Figure5/Figure5A_MotifScanOutput/homerResults/motif4.similar.html]

motif4

## Information for motif4

A
C
T
G
A
G
T
C
G
A
C
T
C
G
A
T
C
G
T
A
C
A
G
T
A
T
G
C
C
G
A
T
  
Reverse Opposite:  

C
G
T
A
A
T
C
G
G
T
C
A
A
C
G
T
C
G
T
A
C
T
G
A
T
C
A
G
T
G
A
C
  

|  |  |
| --- | --- |
| p-value: | 1e-129 |
| log p-value: | -2.990e+02 |
| Information Content per bp: | 1.856 |
| Number of Target Sequences with motif | 1063.0 |
| Percentage of Target Sequences with motif | 35.25% |
| Number of Background Sequences with motif | 7424.4 |
| Percentage of Background Sequences with motif | 16.28% |
| Average Position of motif in Targets | 235.8 +/- 152.9bp |
| Average Position of motif in Background | 204.7 +/- 135.1bp |
| Strand Bias (log2 ratio + to - strand density) | -0.1 |
| Multiplicity (# of sites on avg that occur together) | 1.27 |
| Motif File: | file (matrix) reverse opposite |

### Similar de novo motifs found

|  |  |  |  |  |  |  |  |
| --- | --- | --- | --- | --- | --- | --- | --- |
| Rank | Match Score | Redundant Motif | P-value | log P-value | % of Targets | % of Background | Motif file |
| 1 | 0.895 | C T G A A G T C A T G C A C G T A C G T C G T A A C G T A G T C C G A T | 1e-127 | -294.569395 | 36.34% | 17.22% | motif file (matrix) |
| 2 | 0.935 | T A C G C G T A A C G T G A T C C A G T C G A T C G T A A G C T G T A C C G A T | 1e-126 | -290.558555 | 41.08% | 21.03% | motif file (matrix) |
| 3 | 0.979 | A T G C A C G T A C G T C G T A A C G T A G T C C G A T | 1e-119 | -276.173297 | 36.64% | 17.96% | motif file (matrix) |
| 4 | 0.934 | C G T A A C T G G T C A A C G T C G T A C G T A T C A G T C G A T C G A A G C T A G T C G A C T | 1e-104 | -241.073057 | 26.92% | 11.74% | motif file (matrix) |
| 5 | 0.870 | C G T A C A T G G T C A G C A T G T C A C G T A A T C G T G C A C G A T G A T C G A T C | 1e-73 | -168.423580 | 32.16% | 17.91% | motif file (matrix) |
| 6 | 0.789 | C G T A T C A G G T C A A G C T A C G T A T G C C G A T A C G T G C T A A C G T A T G C C G A T A T G C | 1e-33 | -76.244754 | 27.45% | 18.16% | motif file (matrix) |
